# Progerin expression disrupts critical adult stem cell functions involved in tissue repair

**DOI:** 10.18632/aging.100709

**Published:** 2014-12-21

**Authors:** Laurin Marie Pacheco, Lourdes Adriana Gomez, Janice Dias, Noel M Ziebarth, Guy A Howard, Paul C Schiller

**Affiliations:** ^1^ Research Service and Geriatric Research, Education, and Clinical Center; Bruce W. Carter Veteran Affairs Medical Center; Miami, FL 33125, USA; ^2^ Department of Biochemistry and Molecular Biology; University of Miami Miller School of Medicine; Miami, FL 33136, USA; ^3^ Department of Biomedical Engineering; University of Miami College of Engineering; Coral Gables, FL 33146, USA; ^4^ Department of Medicine; University of Miami Miller School of Medicine; Miami, FL 33136, USA; ^5^ Department of Orthopaedics; University of Miami Miller School of Medicine; Miami, FL 33136, USA

**Keywords:** aging, progerin, HGPS, Hutchinson-Gilford Progeria Syndrome, Progeria, MSC

## Abstract

Vascular disease is one of the leading causes of death worldwide. Vascular repair, essential for tissue maintenance, is critically reduced during vascular disease and aging. Efficient vascular repair requires functional adult stem cells unimpaired by aging or mutation.

One protein candidate for reducing stem cell–mediated vascular repair is progerin, an alternative splice variant of lamin A. Progerin results from erroneous activation of cryptic splice sites within the *LMNA* gene, and significantly increases during aging. Mutations triggering progerin overexpression cause the premature aging disorder Hutchinson-Gilford Progeria Syndrome (HGPS), in which patients die at approximately 13-years of age due to atherosclerosis-induced disease. Progerin expression affects tissues rich in cells that can be derived from marrow stromal cells (MSCs). Studies using various MSC subpopulations and models have led to discrepant results.

Using a well-defined, immature subpopulation of MSCs, Marrow Isolated Adult Multilineage Inducible (MIAMI) cells, we find progerin significantly disrupts expression and localization of self-renewal markers, proliferation, migration, and membrane elasticity. One potential treatment, farnesyltransferase inhibitor, ameliorates some of these effects. Our results confirm proposed progerin-induced mechanisms and suggest novel ways in which progerin disturbs critical stem cell functions collectively required for proper tissue repair, offering promising treatment targets for future therapies.

## INTRODUCTION

Mutations in the gene encoding lamin A lead to numerous disorders, collectively known as laminopathies. Lamin A is localized to the nuclear lamina at the inner-side of the nuclear envelope, contributing to nuclear structural stability and other nuclear functions. Lamin A regulates gene expression by directly binding to DNA, and sequesters heterochromatin and silenced or transcriptionally low genes to the periphery of the nucleus. Some other nuclear functions that are regulated by lamin A include DNA replication, DNA repair, chromatin and nuclear pore complex organization, and gene expression [1-3]. Lamin A is post-translationally farnesylated, yielding immature, pre-lamin A [1, 3-5]. Farnesylation facilitates pre-lamin A integration into the nuclear lamina by virtue of the hydrophobic isoprenoid modification. Following laminar integration, the farnesylated C-terminus is cleaved by the zinc metalloprotease farnesylated-proteins converting enzyme-1 (FACE-1), yielding mature lamin A [6, 7]. Other protein-protein interactions stabilize lamin A in the nuclear membrane. Any splice variant of lamin A that causes truncation and permanent farnesylation results in a mutant of lamin A known as progerin [8, 9]. Progerin is otherwise processed identically to lamin A, but the protein is truncated and permanently farnesylated due to the loss of FACE-1 cleavage site. Progerin expression delocalizes nuclear envelope proteins, disorganizes heterochromatin and nuclear pore complexes, disrupts nuclear morphology, and increases DNA damage and repair [10-13]. Inhibiting farnesylation or knocking down progerin expression with shRNA can ameliorate some of these phenotypes [12, 14-16].

Progerin is expressed in the general population, and its expression significantly increases with age [17, 18]. In aging, the progerin splice variant is produced by the aberrant activation of various cryptic splice sites naturally present within the *LMNA* gene. With age, these cryptic splice sites are erroneously activated at higher rates [10]. Splicing errors observed with increased age are not selective for *LMNA*, but *LMNA* is affected by these age-induced splicing errors. Progerin is also expressed as a result of various genetic mutations that increase activation of the cryptic splice sites in the *LMNA* gene. Mutations leading to progerin overexpression cause a premature aging disorder known as Hutchinson-Gilford Progeria Syndrome (HGPS) [9, 19].

Progerin expression in HGPS patients is most commonly produced by a *de novo* point mutation (C1824T, p.G608G) in exon 11, known as the “classical” HGPS mutation [5, 8, 9]. This silent mutation increases activation of a cryptic splice site, leading to a 50 amino acid deletion near the c-terminal end, wherein the cleavage site for FACE-1 lies. HGPS patients with this classical mutation generally die around 13 years of age, most commonly as a result of atherosclerosis that leads to fatal heart attack or stroke. Progerin (C1824T) is also expressed in atherosclerotic vascular tissues from aged, non-HGPS individuals [18]. HGPS is a severe disorder that disturbs several organ systems leading to hair loss, decreased adipose tissue, increased bone fractures, short stature, vascular stiffness, and severe atherosclerosis. It has been previously recognized that adult stem cell attrition may be a mechanism contributing to these disorders [20-26]. We hypothesize that progerin expression interferes with stem cell functions that are critical in vascular tissue repair. Although many tissues are significantly affected by progerin expression, we focus here on stem cell functions that are relevant for vascular repair. The vascular phenotype in HGPS patients and premature atherosclerosis resulting in death in HGPS patients demonstrate that the vascular compartment is extremely sensitive and responsive to progerin expression.

Because it is difficult to obtain marrow stromal cells (MSCs) from young HGPS patients, previous studies on the effects of progerin expression in MSCs were performed in human telomerase reverse transcriptase (hTeRT) immortalized cells [27]. Forced ectopic hTeRT overexpression can potentially mask progerin effects on self-renewal. Recent advances in cellular re-programming have provided novel induced pluripotent stem cell (iPSC) models of HGPS which have been useful in identifying altered stem cell functions in adult stem/progenitor cells derived from these iPSCs [16, 28]. Each of these models demonstrates unique and distinct perspectives on the effects of progerin expression on stem cell functions.

Here, we evaluate progerin effects on stem cell functions critical to vascular repair using a novel model of a homogenous sub-population of developmentally immature (non-immortalized) MSCs known as marrow-isolated adult multilineage inducible (MIAMI) stem cells. MIAMI cells express various self-renewal markers [29-32] that are not commonly detected in other MSC sub-populations, enabling the unique evaluation of progerin-induced alterations on self-renewal. In addition, MIAMI cells can differentiate into cells that comprise most tissues affected in HGPS, as well as facilitate vasculogenesis and angiogenesis in an *in vivo* mouse model of critical limb ischemia [33]. Because MIAMI cells secrete repair-mediating cytokines, they provide an excellent model for future studies on the mechanisms of previously reported decreases in *in vivo* vascular repair [16]. The MIAMI cell model enables us to evaluate the effects of progerin expression during normal cell and organismal aging in a primary human stem cell population. We focus on self-renewal, proliferation, migration, and membrane flexibility as vital, basic functions that a stem cell population requires in order to participate in more complex processes, particularly proper vascular repair.

## RESULTS

### MIAMI cells express exogenous progerin from a transgene

**Figure 1:**
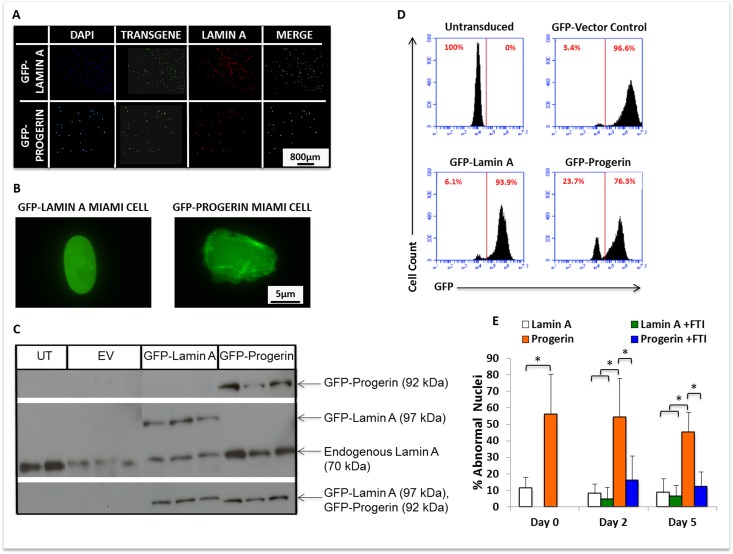
MIAMI cells stably expressing GFP-lamin A and GFP-progerin transgenes demonstrate nuclear abnormalities that can be ameliorated by FTI. **(A)** Immunofluorescent images of GFP-lamin A and GFP-progerin MIAMI cells. MIAMI cells collected from a 20-year old individual were retrovirally transduced with vectors containing GFP-lamin A and GFP-progerin transgenes, and a vector control. Cells were stained with antibody against Lamin A/C and immunofluorescently imaged to visualize Lamin A expression. **(B)** Representative image of MIAMI cell nuclei expressing lamin A only (left) or progerin (right). **(C)** Representative images of western blots probed with antibodies against Lamin A only, Progerin only, and GFP. Endogenous lamin A was used as a loading control. UT=Untransduced MIAMI cells, EV= Empty vector control, Lamin A=GFP-Lamin A transduced, Progerin=GFP-Progerin transduced. Vector control cells in panel C were not transduced with transgene expressing GFP. **(D)** Quantification by flow cytometry of GFP expression in control and transduced cell lines. **(E)** Progerin expression from a transgene significantly increases nuclear abnormalities in MIAMI cells, and FTI treatment ameliorates these effects. FTI treatment did not significantly affect GFP-lamin A MIAMI cells. Values are mean ± standard deviation (n≥3). *p<0.001, calculated by Student's *t*-test.

To investigate the effects of progerin expression on MIAMI stem cell functions, MIAMI cells from a male 20-year old normal donor were retrovirally transduced with GFP-progerin (GFP-progerin MIAMI) cells, GFP-lamin A (GFP-lamin A MIAMI) cells, and a GFP-empty vector control (EV-MIAMI) cells. Transduced cells were selected by GFP^+^ cell sorting, and appropriately express transgenes (Fig. 1A, 1B). To determine the level of transgene expression after selection, we evaluated progerin, lamin A, and GFP protein levels by western blot analyses. Untransduced MIAMI cells were used as a negative control. As expected, endogenous lamin A (70kDa) was expressed in all cell lines, exogenous GFP-lamin A (97kDa) was expressed only in GFP-lamin A MIAMI cells, exogenous GFP-progerin (92kDa) was expressed only in GFP-progerin MIAMI cells, and GFP-tagged proteins (92-97kDa) were detected only in GFP-progerin MIAMI and GFP-lamin A MIAMI cells (Fig. 1C). After sorting, all cell lines were evaluated by flow cytometry to determine the percentage of cells expressing the GFP-transgenes (Fig. 1D).

Progerin expression in MSCs causes nuclear blebs and invaginations, demonstrating decreased structural integrity of the nuclear membrane, as reported by others [1, 13]. To determine whether progerin expression from a transgene in MIAMI cells is sufficient to cause this characteristic phenotype of progerin expressing cells, GFP-progerin and GFP-lamin A MIAMI cells were evaluated for abnormal nuclear morphology using immunofluorescent microscopy (Fig. 1B). Nuclei were scored as abnormal if they contained one or more blebs or invaginations along the membrane. GFP-progerin MIAMI cells expressed significantly higher levels of abnormal nuclei than GFP-lamin A MIAMI cells (Fig. 1E). After blocking farnesylation with farnesyl-transferase inhibitor-277 (FTI-277), the number of abnormal nuclei in GFP-progerin MIAMI cells was significantly reduced (p<0.05) to levels similar to those observed in GFP-lamin A MIAMI cells (Fig. 1E).

**Figure 2:**
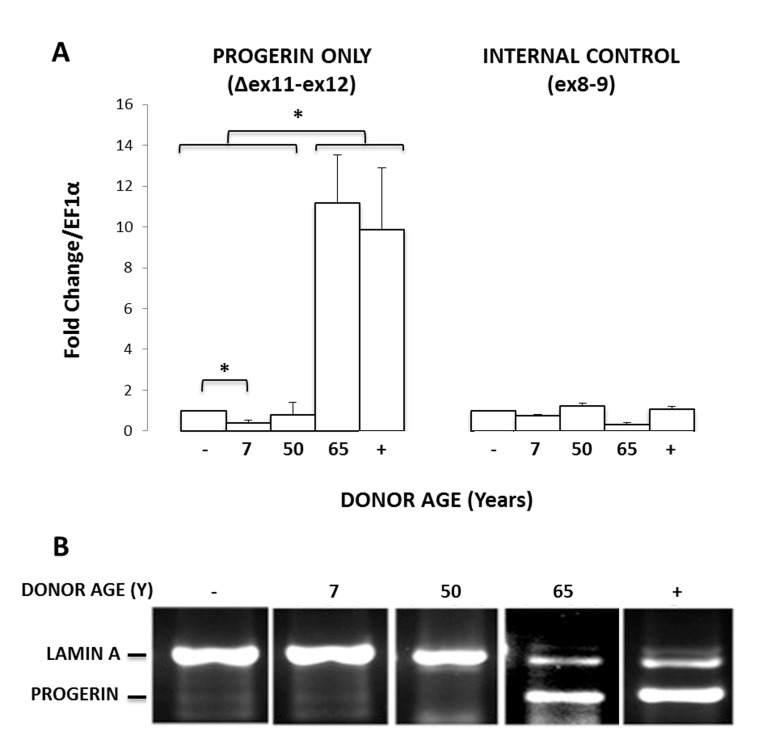
MIAMI cells can express endogenous progerin. **(A)** Endogenous progerin mRNA levels measured by qPCR in MIAMI cells collected from non-HGPS male donors of various ages (7-65 years). Progerin-transduced MIAMI cells collected from a 20-year-old donor (+) were used as a positive control. MIAMI cells collected from a 20 year old (−) were used as a negative control. **(B)** Using primer pairs to amplify both progerin and lamin A, qPCR results were run on an agarose gel to visualize amplification product length. Miami cells collected from a 65-year-old donor and progerin transduced MIAMI cells express both lamin A and progerin products. Values in Panel A are mean ± standard deviation (n≥3). *p<0.05, calculated by Student's *t*-test. P-values indicate significant difference between negative control, unless otherwise noted.

To determine if donor age affects the normal splicing of lamin A in MIAMI cells, we quantified the mRNA levels of progerin (C1824T) transcripts in 5 MIAMI stem cell isolates, collected from non-HGPS male donors ranging in age from 7-65 years old. GFP-progerin MIAMI cells were used as a positive control. To evaluate progerin mRNA levels, we used primer pairs that target progerin only or progerin and lamin A, as previously described [17]. Briefly, the first portion of the forward primer binds before the cryptic splice site (1806), and the second portion of the forward primer binds after the cryptic splice site, enabling the forward primer to only bind if the cryptic splice site has been activated, yielding progerin. To visualize the transcripts, we performed qPCR using a primer pair that binds both lamin A and progerin by binding well outside of, and also spanning, the cryptic splice region. We then ran these products on an ethidium bromide gel. Since progerin is missing 150 base pairs, the two transcripts run as distinctly different lengths. Of those tested, endogenous progerin expression was observed in one MIAMI cell line that was collected from a 65-year old male donor (Fig. 2A and 2B), demonstrating that at least the C1824T cryptic splice site in the *LMNA* gene can be endogenously activated in MIAMI stem cells, particularly in cells isolated from older individuals. Interestingly, these MIAMI cells appear to express progerin mRNA at levels similar to those observed in progerin transduced MIAMI cells.

### Progerin expression alters self-renewal in MIAMI cells

**Figure 3:**
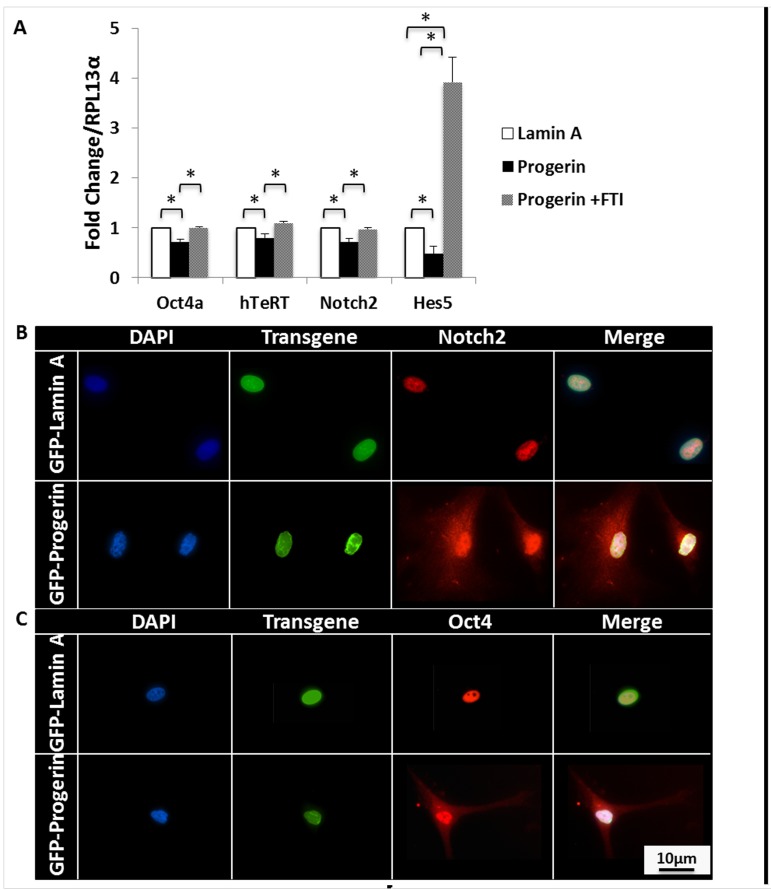
Progerin decreases self-renewal markers and leads to cytoplasmic accumulation of self-renewal transcription factors. **(A)** qPCR analysis of self-renewal markers that are normally expressed in MIAMI cells. Progerin expression significantly decreases Oct4, hTeRT, Notch2, and Hes5 mRNA expression. FTI treatment significantly increases mRNA expression of these self-renewal markers. Dotted line represents control, untreated lamin A-MIAMI cells set to 1. Asterisk over progerin-MIAMI cells signifies progerin-MIAMI cells significantly differently from untreated lamin A-MIAMI cells. Values are mean ± standard deviation (n≥3). *p<0.05, calculated by Student's *t*-test. **(B)** Immunofluorescent images of progerin and lamin A MIAMI cells demonstrate that progerin expression leads to Notch2 and Oct4 accumulation in the cytoplasm, which is normally localized to the nucleus in lamin A MIAMI cells.

To evaluate the effects of progerin expression on MIAMI cell self-renewal, we quantified mRNA of characteristic self-renewal markers highly expressed in MIAMI cells [29-32]. Progerin expression significantly decreased (p<0.05) the total mRNA levels of several of these self-renewal markers, specifically Oct4, hTeRT, Notch2, and Hes5. No effects were observed in the expression of Nanog, Rex1, SSEA4, and CoREST (not shown). When cells were treated with 9μM FTI, the effects of progerin expression on self-renewal mRNA were ameliorated, and most self-renewal mRNA levels in GFP-progerin MIAMI cells significantly increased (p<0.05) to levels similar to those observed in untreated GFP-lamin A MIAMI cells (Fig. 3A).

Notch2 and Oct4 are important transcription factors whose nuclear localization correlates with self-renewal maintenance of undifferentiated MSCs [34-37]. We evaluated spatial organization of Notch2 and Oct4 by immunofluorescent microscopy. Both Notch2 and Oct4 were localized to the nucleus in all control MIAMI cell lines, with little to no detectable expression in the cytoplasm. Interestingly, these transcription factors appeared to be more uniformly dispersed throughout the nucleus and cytoplasm in GFP-progerin MIAMI cells, demonstrating for the first time that progerin expression significantly alters sub-cellular distribution and nuclear localization of transcription factors Notch2 and Oct4 (Fig. 3B).

### Progerin expression decreases cell proliferation

**Figure 4:**
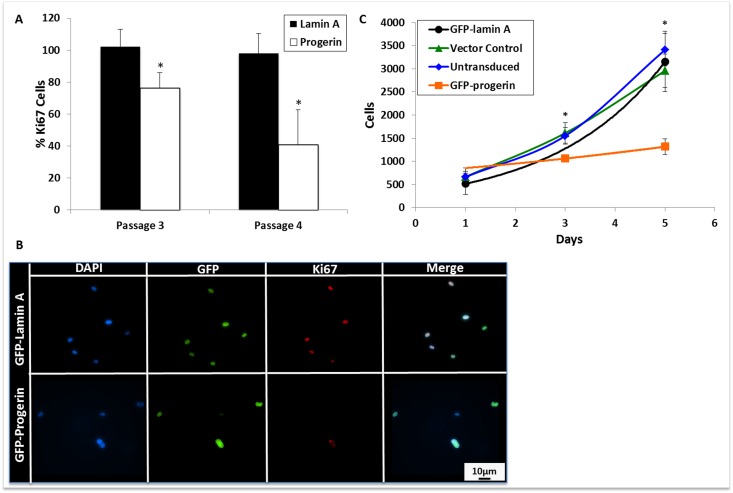
Progerin expression decreases MIAMI cell proliferation. **(A)** Quantification of Ki67 immunofluorescent staining. At least 10 random fields per cell line were selected and cells were scored as positive or negative for Ki67 expression. Progerin expression significantly decreases Ki67 expression when compared to lamin A MIAMI cells in the same passage. **(B)** Immunofluorescent images of transgene and Ki67 expression, a marker of proliferation, in lamin A and progerin MIAMI cells. **(C)** Growth curve of untransduced MIAMI cells, vector control, lamin A, and progerin MIAMI cells. Progerin expression significantly decreases cell number at days 3, and 5 when compared to control cells. There were no significant differences between the control cell lines. Values are mean ± standard deviation (n≥3). *p<0.05, calculated by Student's *t*-test.

To determine whether progerin expression could alter MIAMI cell proliferation, cells were stained with an antibody against Ki67, a marker of cellular proliferation that is expressed in all active phases of the cell cycle, and is not expressed in resting cells [38]. Progerin expression significantly decreased Ki67 expression in MIAMI cells (Fig. 4A and 4B). To further verify progerin effects on cell growth, we quantified cell numbers by employing a colorimetric assay over several days. Progerin expression significantly decreased MIAMI cell numbers, without changes in cell death (quantified by number of detached cells in culture medium) when compared to control MIAMI cells (Fig. 4C).

### Progerin expression decreases cell migration and increases cell stiffness

**Figure 5:**
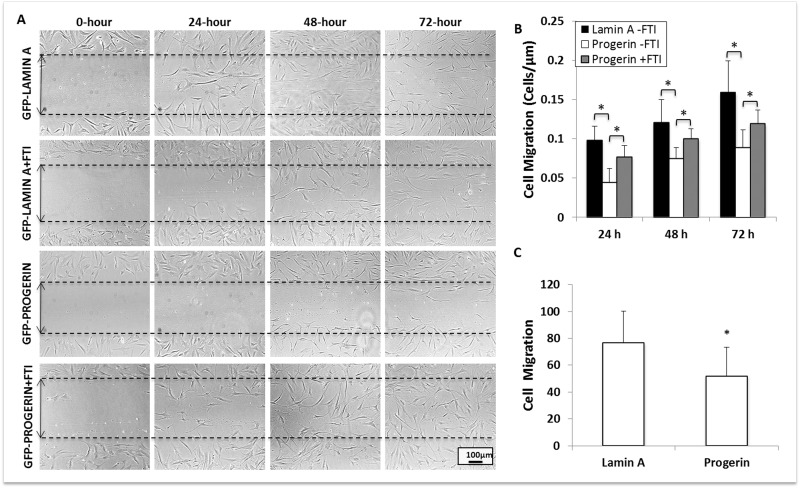
Progerin expression significantly decreases MIAMI cell migration. **(A)** GFP-lamin A MIAMI and GFP-progerin MIAMI cells were scraped at 0 hrs with a 2mL glass pipette tip. Scraped areas were imaged at 0, 24, 48, and 72 hours, with and without FTI, to monitor cellular migration. **(B)** Progerin expression significantly decreases migration at 24, 48, and 72 hours. FTI treatment significantly increases migration at 24, 48, and 72 hours when compared to untreated progerin MIAMI cells. **(C)** Lamin A and Progerin MIAMI cells were plated in MIAMI media containing no FBS in the upper chamber of a transwell. MIAMI media with FBS was placed in the bottom chamber. After 24 hours, cells remaining in the upper chamber were washed away, and cells that migrated to the bottom of the transwell were stained with DAPI and immunofluorescently imaged. Progerin expression significantly decreases migration through the transwell membrane. Values are mean ± standard deviation (n≥3). *p<0.05, calculated by Student's *t*-test.

Cellular migration (i.e., of stem cells to the site of tissue injury) is another crucial aspect of vascular repair. To determine the effect of progerin expression on stem cell migration, we performed a scratch assay on confluent MIAMI cells that express lamin A and progerin. Culture plates with confluent MIAMI cells were superficially scratched with a 2mL glass pipette tip to detach cells from a small, defined area of the cultures. Subsequently, we evaluated cell migration by quantifying the number of cells in the scratched region at 0, 24, 48, and 72 hours after scratching (Fig. 5A). Progerin expression significan-tly decreased MIAMI cell migration when compared to control cells, and these effects persist through 72-hours. Interestingly, FTI treatment significantly increased migration in progerin-expressing MIAMI cells, but did not affect control cell lines (Fig. 5B).

Additionally, we confirmed the effects on cell migration using a transwell assay, in which MIAMI cells expressing lamin A or progerin were plated in the upper chamber of a transwell system in MIAMI media containing no FBS. MIAMI media containing 3% FBS was placed in the bottom chamber. After 24 hours, cells that had migrated through the transwell membrane were fixed, DAPI stained, and manually counted. The control cells (untransduced, vector control, and GFP-lamin A MIAMI cells) migrated through the membrane significantly more than GFP-progerin MIAMI cells (Fig. 5C).

Because lamin A interacts with cytoskeletal components such as actin [39-41], we examined whether progerin-induced nuclear membrane alterations could cause cyto-skeletal changes leading to increased cellular membrane stiffness, as a potential mechanism for decreased migration (especially as seen in the transwell assay).

Using atomic force microscopy, the Young's modulus of elasticity, or membrane stiffness, was evaluated. By quantifying applied force to the cell membrane at cytoplasmic and nuclear positions on the cell surface, we measured regional differences in stiffness.

**Figure 6:**
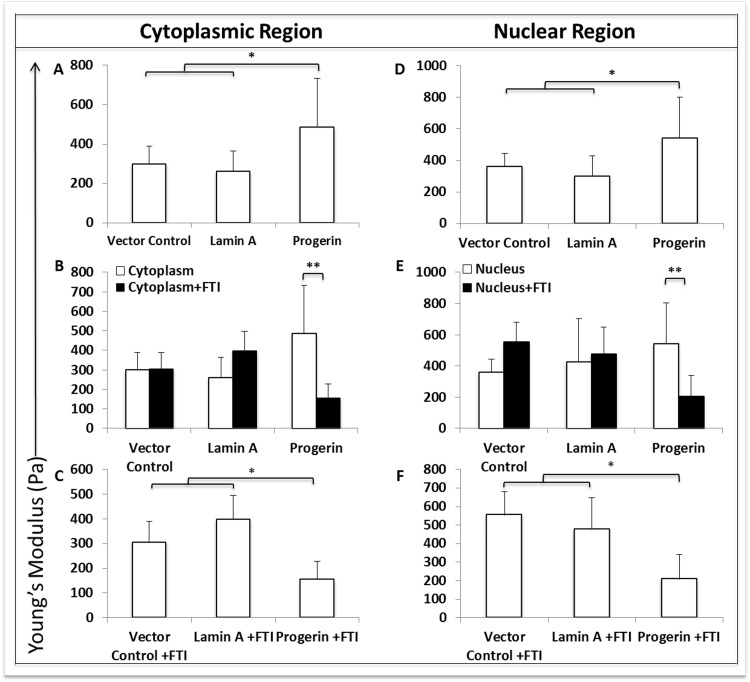
Progerin expression significantly alters membrane stiffness when measured by atomic force microscopy. **(A,D)** Progerin expression significantly increases membrane stiffness in both cytoplasmic **(A)** and nuclear **(D)** regions when compared to control cell lines, while stiffness in cytoplasmic and nuclear regions are not significantly different between control cell lines. **(B, E)** FTI treatment significantly decreases membrane stiffness in cytoplasmic **(B)** and nuclear **(E)** regions in progerin-MIAMI cells when compared to untreated progerin-MIAMI cells, while stiffness in cytoplasmic and nuclear regions are not significantly different before and after FTI treatment in control cell lines. **(C, F)** FTI treatment significantly decreases membrane stiffness in cytoplasmic **(C)** and nuclear **(F)** regions in progerin-MIAMI cells to levels that are significantly less than treated control cell lines, while stiffness in cytoplasmic and nuclear regions are not significantly different between control cell lines after FTI treatment. Values are mean ± standard deviation (n≥3). *p<0.05, **p<0.01, calculated by Student's *t*-test.

Progerin expression significantly (p<0.05) increased stiffness in both cytoplasmic (Fig. 6A) and nuclear (Fig. 6D) regions of MIAMI cells compared to control MIAMI cells. Importantly, FTI treatment significantly (p<0.005) decreased stiffness in both cytoplasmic (Fig. 6B) and nuclear (Fig. 6E) regions in GFP-progerin MIAMI cells when compared to untreated GFP-progerin MIAMI Cells. FTI treatment did not significantly alter stiffness in control cells (Fig. 6B, 6E). Interestingly, FTI treatment decreases stiffness in both cytoplasmic (Fig. 6C) and nuclear (Fig. 6F) regions in GFP-progerin MIAMI cells to levels that are significantly less than treated control cell lines (p<0.05).

## DISCUSSION

The vascular system is under constant mechanical and inflammatory stress. Fluid pressure and sheer stress combined with inflammatory cytokines lead to damage of the arterial compartment primarily, resulting in injury and death of endothelial, vascular smooth muscle cells and pericytes in the arterial and arteriole walls. In order to repair injured arteries and maintain vascular integrity, damaged or dying cells need to be replaced in a rapid and efficient manner. This is achieved by progenitor or stem cells sensing the damage, migrating to the injured area, differentiating into the needed cell phenotype, and modulating the inflammatory milieu at the injury site. Furthermore, sufficient numbers of these cells are needed in order to maintain a vascular reparative capacity throughout adult life. Thus, these stem/progenitor cells need to self-renew and proliferate in order to maintain a suitable pool of cells available for repair.

The arterial compartment is extremely sensitive to progerin expression, demonstrated by the robust atherosclerosis and vascular diseases exhibited by HGPS patients. Progerin is also expressed in atherosclerotic vascular tissues collected from aged, non-HGPS individuals [18]. Both cases indicate a mechanistic role for progerin expression in interfering with general vascular tissue homeostasis. Efficient vascular repair that is unimpaired by disease or aging requires an adult stem cell population that can maintain their immature status (self-renewal), proliferate, detect damaged tissue and migrate toward it, and contribute to tissue repair by decreasing inflammation and differentiating into necessary cell lineages. We have shown that MIAMI cells can perform all these described functions and participate in the repair of the arterial compartment both *in vivo* and *in vitro* [27-31]. Interrupting any of these key stem cell functions could decrease vascular repair, increase persistent vascular damage, and result in atherosclerosis and eventual vascular accidents.

The results presented here demonstrate that progerin protein interferes with basic, critical stem cell functions that play an essential role during vascular repair. Endogenous progerin expression observed in MIAMI cells collected from a non-HGPS older donor suggest that MIAMI cells can accumulate progerin *in vivo*, and therefore are likely subject to the effects of progerin expression. One remarkable observation is that progerin mRNA in MIAMI cells from an aged (65-year old) donor appears to be expressed at similar levels when compared to transduced GFP-progerin MIAMI cells. Because it is likely that cells from aged individuals express progerin at lower levels than cells from HGPS patients [18], we consider our transduced GFP-Progerin MIAMI cells provide a suitable model to assess the effects of progerin expression in the context of physiological aging in a defined stem/progenitor cell population, with implications to age-related disorders during organismal aging.

Overexpression of lamin A can lead to a less severe progerin-like phenotype [27]. Because MIAMI cells already contain two copies of the wild-type lamin A gene, inserting the GFP-progerin transgene not only introduces progerin expression, but also increases absolute lamin A expression. Therefore, it is important to address any discrepancy in gene copy number. In our experiments, we normalized to lamin A transduced MIAMI cells to account for the additional third copy of the gene, either mutant or wild-type. Even when compared to cells that are expressing total lamin A at relatively equivalent levels, progerin expression still significantly affects several specific MIAMI stem cell functions that are directly relevant to physiological aging.

By reducing hTeRT levels, we expect that progerin expression promotes telomere length shortening in affected adult stem cells. This could contribute to their premature depletion. In contrast, in other MSC models of progeria where MSCs were immortalized by hTeRT forced overexpression, this effect was not evident [27], suggesting an advantage of this MIAMI cell model and demonstrating a novel finding using our system. It has been reported that shortened telomeres trigger non-specific, global erroneous splicing. The erroneous splicing triggered by shortened telomeres increased the activation of cryptic splice sites within the *LMNA* gene, thus increasing progerin expression, and suggesting that progerin may actually up-regulate expression of itself during aging by shortening telomeres in adult stem cells [10]. Decreased hTeRT in progerin-expressing MSCs could interfere with vascular repair by promoting cellular senescence, however, assessing the role of endogenous progerin expression on stem cell functions as they relate to vascular repair are beyond the scope of the studies presented here.

Gene expression of hes5, a downstream effector of Notch 2, is also decreased by progerin expression. Interestingly, FTI treatment increased Hes5 expression to levels that are significantly higher than Hes5 expression observed in control cells. Because FTI-277 is non-specific [42, 43], we expect that some cellular functions may be more dramatically affected than others, depending on their interactions with and regulation by other farnesylated proteins. FTI treatment restores transcript levels of almost all self-renewal markers that were decreased by progerin expression to levels similar to controls (Fig. 3A). It is encouraging to see that FTI treatment has a positive effect on self-renewal, and this data suggests a novel mechanistic role of FTI treatment in HGPS clinical trials.

Another interesting finding is the progerin-induced alterations in the nuclear localization of transcription factors Oct4, important to MSC self-renewal [36, 37], and Notch2, key to cell signaling in stem cell self-renewal and differentiation [34, 44]. By reducing the expression and nuclear localization of Notch2 and Oct4, progerin expression could interfere with vascular repair by reducing proliferative capacity, possibly resulting in depletion of the circulating stem cell population by suppressing self-renewal programs and proliferation while potentially promoting stem cell maturation. Altering the self-renewal profile would likely prevent the repair of vascular damage by depleting the MSC population available, which heavily participates in the repair cascade that is vital to vascular repair.

Proliferation analysis and Ki67 staining demonstrates that progerin-expressing MIAMI cells proliferate at a significantly lower rate than MIAMI cells that express only lamin A at equivalent levels. With a dwindling population due to slowed proliferation combined with decreased self-renewal, adult MIAMI cells that are directly involved in vascular repair [33] are not only functionally compromised, but depletion of such population could exacerbate their inefficiency.

MIAMI cells and other MSC populations play a critical role in endogenous tissue repair *in vivo* because they secrete abundant cytokines and growth factors [33, 45]. To produce these reparative effects, MSCs must home to, or migrate towards, the site of tissue damage [33, 46, 47]. Decreased migration due to progerin and amelioration of these effects by FTI demonstrate that the permanent farnesylation of progerin plays a valuable role in MSC mobility. Cellular migration is a critical repair-mediating stem cell function, enabling stem cells to move toward, interact with, and repair damaged tissues. By decreasing MIAMI cell migration, progerin expression poses a threat to vascular tissue repair, because progerin-expressing MIAMI cells will interact less with damaged tissues. By restoring migration, FTI treatment may potentially enable more effective tissue repair in the presence of progerin by enabling MSC production of anti-inflammatory and migration-inducing cytokines in the microenvironment of regions of vascular damage.

Cellular migration is driven by actin-polymerization at the leading edge of the cell, thus requiring unrestricted intracellular movement of actin molecules [48]. Because lamin A interacts with cytoskeletal components such as actin [39, 40], effects of the progerin mutant on membrane stiffness and elasticity could, in part, explain decreases in cellular migration. Lamin A expression has been reported to positively correlate with tissue and matrix stiffness, and dominates over lamin B expression in MSCs [49]. Here, we explore the role of a lamin A mutant, progerin, in MSC membrane stiffness, and demonstrate that progerin modulates mechanical properties that appear central to cell migration. The membrane stiffness measurements we performed are dependent on all cellular components located beneath the cantilever tip. Therefore, cytoplasmic membrane stiffness also contributes to the stiffness reported for nuclear regions. Although the nuclear stiffness is not directly measured here, changes in nuclear stiffness are reflected in these measurements, as have been reported in other MSC populations [50], suggesting that both nuclear and cytoplasmic stiffness may be altered by progerin expression. Thus, our findings would suggest that tissue and matrix stiffness, as reported by Swift et al. [49], would be further increased in correlation to progerin expression levels.

Increased membrane stiffness provides a possible explanation for our observed decreased cellular migration. As membrane stiffness increases, cellular motility may be compromised. In addition to compromising cellular migration during vascular repair, altered membrane stiffness may interfere with cellular architecture that is required for proper MSC function. Membrane stiffness is decreased so significantly by FTI that treated GFP-progerin MIAMI cells are more elastic than treated control cells. It has been reported that permanent farnesylation of progerin is not exclusively responsible for the progerin phenotype, and the 150 base pair deletion also plays a [less significant] role [51]. Because FTI-treated GFP-progerin MIAMI cell membranes are more elastic than controls, we consider that the 150 base pair truncation may also play a role in progerin-mediated membrane stiffness. Lamin A contains several binding sites for actin, at least one of which is lost with progerin truncation [40]. Permanent farnesylation of progerin could mask the loss of this actin binding site due to the tight interaction between the progerin-farnesyl group and nuclear envelope [52-54] and/or gained binding partners by progerin mutant [2]. Blocking farnesylation of progerin loosens the interaction between unfarnesylated progerin and the nuclear envelope [52-54], suggesting that the effects of the lost progerin-actin binding site is masked by the tight farnesyl-nuclear envelope interaction observed in permanently farnesylated progerin.

It has been reported that progerin expression *in vitro* has little to no effect on osteogenic differentiation, but does increase stress-induced senescence in iPSCs generated from HGPS fibroblasts [16]. Conversely, alterations in osteoblastogenesis and bone metabolism have been reported as a consequence of altered lamin A/C levels *in vitro* and *in* vivo [55-57]. Mechanistic studies in our lab strongly suggest that progerin expression in MIAMI cells alters their osteoblastic differentiation program (manuscript in preparation). Moreover, a recent study strongly suggests a critical role of PARP1 in mediating smooth muscle cell loss in HGPS patients [26].

Together, our results demonstrate a farnesyl-dependent mechanism in which progerin expression interrupts complex stem cell processes by inducing structural alterations in the nucleus that interfere with critical, basic stem cell functions that are required for vascular repair: self-renewal, proliferation, membrane fluidity and cell migration. Progerin-induced alterations of these functions are likely to correlate with a diminished capacity for vascular repair by these cells *in vivo* which would have a significant relevance to organismal aging. Furthermore, FTI treatment ameliorates some effects of progerin, revealing mechanistic processes that are highly significant to clinical applications of FTI treatment in HGPS patients. However, not all progerin-induced alterations in stem cell functions may be ameliorated by FTI treatment. Additionally, FTI treatment may alter specific stem cell functions involved in tissue repair independently of progerin expression, such as modulation of the local inflammatory environment. Thus, additional studies are warranted to increase our understanding of progerin-induced alterations of stem cell functions. These data strongly support progerin effects on hMSC functions by demonstrating novel mechanisms (e.g., membrane stiffness, self-renewal, subcellular localization/traffic-king) of progerin-induced alterations on hMSC functions required for effective vascular tissue repair.

## METHODS

### MIAMI cell culture isolation and propagation

MIAMI cells were isolated from whole bone marrow of a 20-year old (H3515) living male donor (MIAMI #H3515, Lonza, Basel, Switzerland; [30]). We have also used bone marrow from vertebral bodies of cadaveric donors of different ages and gender obtained from the University of Miami Tissue Bank and other sources after Insititutional Review Board (IRB) approval. Briefly, whole bone marrow was plated (without prior centrifugation, immunoselection, or depletion) at 10^5^ cells/cm^2^ on fibronectin-coated (10ng/mL) plates in a low oxygen (3%) using MIAMI cell medium (see below). After seven days, non-adherent cells were carefully removed and half the medium was replaced. After seven days, the complete medium of adherent cells were carefully replaced with fresh medium. All experiments were performed with H3515 cells unless otherwise stated. MIAMI cells were grown on 10ng/mL fibronectin (Sigma, F2518) coated vessels at 37°C, 3% oxygen [30]. MIAMI media was composed of DMEM-low glucose media (#11885, Gibco, Grand Island, NY, USA,), 3% fetal bovine serum (FBS; Lot 66310, Hyclone, Waltham, MA USA, lot selected for maintaining MIAMI cell characteristics), 20mM ascorbic acid (#49752, Sigma, St. Louis, MO, USA), an essential fatty acid solution (#A9673, Sigma), and 1:1000 antibiotics (100 U/mL penicillin, 0.1 mg/mL streptomycin; #15140, Gibco). To passage, MIAMI cells were removed with 0.25% trypsin/EDTA, centrifuged at 400× g for 5 minutes at 4°C, counted with 0.4% trypan blue on a hemocytometer, and then plated at a density of 100-500 cells/cm^2^.

### Retroviral Transduction

293T cells were plated at 50 × 10^3^ cells/cm^2^ in T75 flasks. Expansion media was composed of DMEM-high glucose media (#11995, Gibco), containing 10% FBS (Lot 66310, Hyclone), and 1% antibiotics (#15140, Gibco). On day 1, 293T cells were transfected with 41μL Fugene6 (E2691, Promega, Madison, WI, USA), 6 μg lentiviral packaging vector (pUMVC; #8449, Addgene, Cambridge, MA, USA), 600 ng envelope vector (pVSVG; #8454, Addgene), and target vectors: GFP-progerin-pBABE (#17663, Addgene), GFP-lamin A-pBABE (#17662, Addgene), and GFP-pBABE (#10668, Addgene) in antibiotic-free 293T media, per instructions. On day 2, viral supernatants from 293T cells were discarded and replaced with MIAMI media. H3515 MIAMI cells were seeded at 500-800 cells/cm^2^ in MIAMI media, modified to contain 10% FBS (to boost overnight proliferation). On days 3-4, MIAMI media on MIAMI cells was replaced with viral supernatants from transfected 293T cells. Viral supernatants were centrifuged at 400× g for 5 minutes at 4°C, filtered through 0.45μm syringe filter (4614, Pall, Port Washington, NY, USA), and supplemented with 10μg/mL protamine sulfate (P2162, Sigma). On day 5, viral supernatant from infected MIAMI cells was discarded and replaced with fresh MIAMI media. Cells were incubated in 3% O_2_ at 37°C until 50-70% confluent. Transduced cells were selected by GFP-cell sorting, and re-seeded for expansion at 500 cells/cm^2^.

### GFP-cell sorting

At 50-70% confluence, transduced cells were removed, re-suspended in 1000 cells/μL phosphate buffered saline (PBS), and filtered through 35μm strainer capped-test tubes (#352235, BD Falcon, San Jose, CA, USA). Untransduced H3515 MIAMI cells were used as a negative control. After sorting, cell suspension was mixed 1:1 with antibiotics (#15140, Gibco) by gentle pipetting, centrifuged at 400× g for 5 minutes at 4°C, and re-seeded at 500 cells/cm^2^.

### Western blotting

Cells were collected and lysed using NP40 lysis buffer for 30 minutes on ice with constant agitation. Lysates were centrifuged at 12,000 x g for 5 minutes at 4°C, supernatants were collected, and then protein content was quantified (#500-0006, Biorad, Hercules, CA, USA). Protein was separated on 10% polyacrylamide gels and transferred to nitrocellulose membranes. The membranes were blocked with 5% milk, then probed with primary antibodies against Lamin A (MAB3540, Millipore, Billerica, MA, USA), Progerin (#05-1231, Millipore), GFP (AB3080, Millipore), Oct4 (MAB4401, Millipore), and Notch2 (Ab8926, Abcam, Cambridge, MA, USA) in 5% milk for 1h at room temperature. Membranes were then washed in TBS-tween and incubated in 5% milk supplemented with HRP-linked secondary antibodies (SC2005, goat anti-mouse IgG-HRP; SC2060, goat anti-mouse IgG1-HRP; SC2004, goat anti-rabbit IgG-HRP; SC2020, donkey anti-goat IgG-HRP, Santa Cruz Biotechnology, Dallas, Texas, USA). Protein bands were detected by enhanced chemiluminescence using the ECL Plus kit (RPN2132, GE Life Sciences, Pittsburg, PA, USA). Total protein stained with coomassie brilliant blue (Biorad, 161-0400) was used as a loading control.

### Real time PCR (qPCR)

RNA was collected from cells using an RNAqueous RNA isolation kit (AM1912, Life Technologies, Grand Island, NY, USA) and precipitated for at least 12 hours at −80°C. RNA precipitate was re-suspended in 15μL elution buffer and then quantified using a Nanodrop spectrophotometer. cDNA was synthesized and suspended to a final concentration of 10ng/μL. qPCR was performed with Brilliant II Sybr Green PCR Master Mix (#600834, Agilent Technologies, Santa Clara CA, USA) using 100ng cDNA per reaction. qPCR results were analyzed and gene expression was normalized to EF1α and/or RPL13α [58].

### Cell Proliferation Analysis

The colorimetric assay CellTiter 96® AQueous One Solution Cell Proliferation Assay (G3580, Promega) was used to evaluate proliferation. MIAMI cells were plated at 500 cells/cm^2^ (8 wells per cell line). On the day of analysis cells were incubated with 100μL MIAMI media supplemented with 20μL Promega celltiter solution at 37°C for 1-4h. Miami media:Promega celltiter solution was used as a blank. Plates were read at 490nm.

### Farnesyltransferase Inhibitor (FTI) treatment

10μM farnesyltransferase inhibitor 277 (FTI-277; F9803, Sigma-Aldrich) was added to MIAMI media when cells reached 50% confluency. Treatment lasted at least 4 days, and media was changed every other day.

### Immunocytochemistry

MIAMI cells were fixed in 4% paraformaldehyde for 15 minutes at room temperature, rinsed 3× 5 minutes in PBS, and stored in sterile water at 4°C. Cells were permeabilized with 0.1% triton-x for 5-10 minutes at room temperature, blocked in 3% PBS-BSA for 30 minutes at room temperature and then stained with primary antibodies against GFP (AB3080, Millipore), Notch 2 (Ab8926, Abcam), Oct4 (MAB4401, Millipore), Lamin A (MAB3540, Millipore), Progerin (#05-1231, Millipore), Ki-67 (KI67-MM1-L-CE, Leica Biosystems, Buffalo Grove, IL,USA) for 1h at room temperature. Rinsed cells were incubated with fluorescently labeled secondary antibodies for 1h at room temperature. Coverslips were mounted on glass slides using DAPI mounting media. Cells not plated on coverslips were incubated with DAPI antibody for 15 minutes at room temperature prior to imaging.

### Transwell Migration Assay

MIAMI cells (1.5-2.5×10^4^) were suspended in 100μL MIAMI media containing no FBS, and seeded into transwell inserts (8μm pore size membrane; #3422, Corning Costar, Tewksbury MA, USA). The transwell inserts were placed into 24-well plates containing 400μL of MIAMI media containing 3% FBS and incubated at 37°C, 3% O_2_ for 22 hours. The transwells were removed from the 24-well plates, and remaining cells were wiped from the top of the membrane. The bottom side of the membrane was fixed with 4% paraformaldehyde for 15 minutes at room temperature and stained with DAPI. Cell migration was measured by counting all DAPI-stained cells present on the bottom of the transwell membrane using a fluorescence microscope.

### Scratch Assay

MIAMI cells (20,000 cells/cm^2^) were seeded in 12 well plates and were allowed to attach overnight at 37°C, in 3% O_2_. After 24-hours (cells were approximately 100% confluent) a 2mL pipette tip was continuously scraped across the diameter of the well, creating a clear lesion in which no cells were present. Cells were washed twice before adding fresh MIAMI media.

### Atomic Force Microscopy

MIAMI cells (1,000 cells/cm^2^) were seeded in MIAMI media in 35mm x 10mm culture dishes (Sarsdedt). Cells were incubated at 37°C, 3% O_2_ until approximately 50% confluent. Elasticity was then measured using a custom-built nanoindenter [50, 59]. A pyramidal, AFM cantilever tip (0.01N/m, silicon nitride, 20nm tip radius, MLCT series, Bruker AFM Probes, Camarillo, CA, USA) was used to indent the cell. The measurements were conducted using a cantilever approach and retraction speed of 1.5μm/s and a maximal indentation force of 60nN. The recordings were repeated at least 15 times per cell in both the nuclear and cytoplasmic regions. The Bilodeau model for a pyramidal indenter was used to calculate Young's modulus of elasticity. For FTI-treated cells, drug in FBS-free media was added when cells were approximately 50% confluent. Media was changed every other day for four days, at which time the elasticity was measured. Cells were washed and placed in DMEM during measurements.

### Statistical Analysis

All data sets were tested for significance using Student's *t-*test (two-tailed, two sample, unequal variance). Statistical significance was determined by *P*-values less than 0.05, unless otherwise noted.
